# Physicochemical Properties and Sensory Acceptability of a Next-Generation Functional Chocolate Added with Omega-3 Polyunsaturated Fatty Acids and Probiotics

**DOI:** 10.3390/foods10020333

**Published:** 2021-02-04

**Authors:** Paulinna Faccinetto-Beltrán, Andrea R. Gómez-Fernández, Norma E. Orozco-Sánchez, Esther Pérez-Carrillo, Luis Martín Marín-Obispo, Carmen Hernández-Brenes, Arlette Santacruz, Daniel A. Jacobo-Velázquez

**Affiliations:** 1 Tecnologico de Monterrey, Escuela de Ingeniería y Ciencias, Av. General Ramón Corona 2514, Zapopan C.P. 45201, Jal, Mexico; paulinnafb1995@gmail.com (P.F.-B.); andrea.rebeca.gf@gmail.com (A.R.G.-F.); 2 Escuela Mexicana de Confitería y Chocolatería, Melchor Ocampo 926, San Luis Potosi C.P. 78280, SLP, Mexico; ccinvestigacionc@gmail.com; 3 Tecnologico de Monterrey, Escuela de Ingeniería y Ciencias, Av. Eugenio Garza Sada 2501, Monterrey C.P. 64849, NL, Mexico; perez.carrillo@tec.mx (E.P.-C.); lmmarin@live.com.mx (L.M.M.-O.); chbrenes@tec.mx (C.H.-B.); asantacruz@tec.mx (A.S.)

**Keywords:** milk chocolate, ω3 PUFAs, probiotics, cognition, physicochemical properties

## Abstract

In this study, a milk chocolate formulation was developed to serve as vehicle of Omega-3 (ω3) polyunsaturated fatty acids (PUFAs) and probiotics (*L. plantarum* 299v and *L. rhamnosus* GG). Fish oil (FO) was incorporated in chocolate as a source of ω3 PUFAs. Probiotics (Prob) and FO were added during tempering, obtaining chocolates with 76.0 ± 5.2 mg (FO1) or 195.8 ± 6.5 mg (FO2) of ω3 PUFAs, and >1 × 10^6^ CFU of Prob per chocolate portion (12 g). The physicochemical properties (rheological analysis, texture, surface instrumental color, $a_{w}$, and fatty acid profile), and sensory acceptability of the formulations were determined. Prob and FO generated a decrease in L* and white index (WI) values. Except for Prob + FO2, all treatments showed a decrease in $a_{w}$. Rheological parameters of FO1 and Prob + FO1 presented the most similar behavior as compared with the control. Prob or FO1 addition did not affect the overall consumer’s acceptability of chocolate; and when both nutraceuticals were combined (Prob + FO1) the product showed adequate overall acceptability. FO2 formulations were not considered adequate to maintain physicochemical properties and sensory acceptability of chocolate. Results indicated that milk chocolate is a suitable vehicle for delivering ω3 PUFAs and Prob, which are essential to enhance cognitive development in children.

## 1. Introduction

Today, society seeks new eating habits and lifestyles to improve their life expectancy. These new eating habits can help to prevent various diseases such as diabetes, cardiovascular disease, obesity, Alzheimer’s, Parkinson’s, etc. For this reason, the food industry has strived to develop next-generation functional foods that can meet these demands. Next-generation functional foods added with bioactive ingredients have high potential because they can reduce chronic degenerative diseases while providing proper nutrition [1,2]. Likewise, much research focuses on identifying functional food ingredients as product fortifiers to provide health benefits beyond the nutritional value. These bioactive compounds are those ingredients that, when added to food, give an improvement in health. The main bioactive ingredients, also known as nutraceuticals, which have been used over time, are prebiotics, probiotics (Prob), amino acids, proteins, omega-3s (ω3) polyunsaturated fatty acids (PUFAs), plant extracts, antioxidants, and vitamins, among others. The food industry has obtained favorable results in the development of new products fortified with nutraceuticals ingredients. Likewise, it is estimated that globally the bioactive compounds to be used in the most significant proportion in the following years are fibers, vitamins, plant extracts, carotenoids, probiotics, and ω3 PUFAs [3].

The incorporation of bioactive ingredients such as ω3 fatty acids in children diet is essential because they play a crucial role in cognitive development [4]. Fatty acids of n-3 family, such as docosahexaenoic acid (C22:6, DHA) and eicosapentaeonic acid (C20:5, EPA), are fundamental for early-stage development and growth. These fatty acids’ deficiencies affect brain performance, inducing behavior and cognitive impairments [5]. Likewise, it has been shown that there is a relationship between the cognition of the brain with peripheral functions through the interaction of the central nervous system in the brain and the enteric nervous system in the intestine [4]. Therefore, targeting gut microbiota is essential for the development of strategies for health care. In this context, Prob have potential in the treatment of neurological problems [4]. Likewise, it has been found that the addition of lactic acid probiotic bacteria in functional products helped improve cognitive function in Alzheimer’s patients [6]. In addition, the decrease of probiotic strains, such as *L. plantarum* have been associated with cognitive impairment [7]. For that reason, the addition of probiotic strains such as *L. plantarum* 299v has been found to improve cognitive functioning in patients with major depression [8]. Additionally, other lactic bacteria such as *L. rhamnosus* GG have shown improvements in children’s and adolescents’ cognitive function, generating the reduction of behavior and communication disorders such as autism [9]. Therefore, the development of next-generation functional foods is crucial to make available to consumers products with beneficial health effects [10].

The development of new products containing bioactive ingredients can be challenging because various parameters need to be considered, such as the vehicle, the compound’s safety, product stability, and possible interactions between the ingredients [3]. The acceptance of these foods depends mainly on the vehicle type used to carry the main compound [10]. Chocolate, combined with a healthy lifestyle, has been considered a functional food [11]. For this reason, it has been found that milk chocolate can function as a vehicle for easy and efficient administration of compounds as it has been confirmed to be suitable for carrying both ω3 PUFAs and probiotics [12,13,14,15].

Many studies have revealed that price and taste are essential factors that determine the acquisition of functional foods. It has been found that consumers are not able to compromise the flavor despite the benefits that a product could provide [12]. The quality of chocolate is established during the production process and can be monitored with variables such as viscosity, texture, and moisture, which are factors that influence sensory characteristics [16].

In the present study, probiotics (*L. plantarum* 299v and *L. rhamnosus* GG), were microencapsulated by spray-drying, using maltodextrin and sodium alginate as encapsulating agents; and the effect of fish oil (rich in ω3 PUFAs) and microencapsulated Prob addition either alone or combined, on the physicochemical characteristics and sensory acceptability of milk chocolate were evaluated. Fish oil (FO) was added at two different concentrations (3.24% or 6.48% *w*/*w*) in chocolate formulation either alone or combined with microencapsulated Prob.

## 2. Materials and Methods

### 2.1. Chemicals and Active Ingredients

For chocolate bars elaboration, milk-chocolate pellets 33% cocoa (Vanleer, Barry Callebaut, Zurich, Switzerland) were used. The FO rich in omega-3 PUFAs (Omega Pure^®^) was purchased from America Alimentos S.A. de C.V. (Zapopan, Jalisco, México). Probiotic strains *Lactobacillus plantarum* 299v (DSMZ 9843) and *Lactobacillus rhamnosus* GG (ATCC 7469) were obtained from German Collection of Microorganisms and Cell Cultures (DSMZ, Braunschweig, Germany) and American Type Culture Collection (ATCC, Manassas, VA, USA), respectively. Sodium alginate was purchased from Deiman (Guadalajara, Jal, México) and maltodextrin food grade from Best Ingredients México (Monterrey, NL, México). Xylose Lysine Deoxycholate (XLD) Agar, Tetrathionate Broth Base, Rappaport Vassiliadis Broth, and VRBA agar and MRS agar (BD Difco^TM^) were obtained from Sigma-Aldrich^®^ (St. Louis, MO, USA). Methanol, sulfuric acid, toluene and hexane (GC grade) were acquired from CTR Scientific (Monterrey, NL, México). The undecanoic acid standard and fatty acid mixtures standard GLC 566 (39 fatty acid methyl esters) were purchased from Nu Chek Prep Inc. (Elysian, MN, USA).

### 2.2. Probiotic Microencapsulation and Viability Determination

To maintain cell viability, probiotics were subjected to spray-drying microencapsulation using a spray-dyer (ADL 311S, Yamato Scientific Co., Ltd., Santa Clara, CA, USA). An inoculum of each probiotic strain was prepared in MRS broth and incubated for 24 h at 35 °C. After incubation, the inoculated medium was transferred into 50 mL plastic tubes and centrifuged (10,000× *g*, 10 min). The pellet was recovered and resuspended with 30 mL of peptone water (0.1% peptone, 0.85% NaCl, pH 7), centrifuged (10,000× *g*, 10 min) and the supernatant was discarded. This procedure was repeated twice and resuspended in a final volume of 30 mL of peptone water. Before spray-dying, the suspended cells were added to 750 mL of microencapsulation mix (maltodextrin 10% *w*/*v*, sodium alginate 2% *w*/*v*). The microencapsulation conditions used in the spray-drier had an inlet temperature of 130 °C, outlet temperature 60 °C, blower = 7, pump = 1.5, and pressure 0.13 Pa. Microencapsulation yield was calculated as 24.21%. After spray-drying, probiotic viability was determined by homogenizing 0.1 g of microencapsulated powder with 9 mL of peptone water (1:9 dilution). Serial dilutions were prepared, and 100 µL of diluted samples were added into the MRS agar plates by duplicate and incubated for 24 h at 35 °C (Shel lab 1535, VWR, Randor, PA, USA).

### 2.3. Milk Chocolate Production and Determination of Probiotics Viability

Chocolate pellets were melted in a water bath at 43 °C in a stainless-steel container. Once it was fully melted and homogenized, the chocolate was tempered to 29 °C using a marble plate. The chocolate was then transferred to the stainless-steel container and returned to the water bath until reaching 31 °C. Fish oil and the microencapsulated probiotics mix were added during the tempering process according to each treatment. The amount of powder containing microencapsulated probiotics added to milk chocolate formulation was calculated in order to obtain >1 × 10^6^ CFU of probiotics per portion (12 g) of chocolate, whereas fish oil was added either at 3.24% *w/w* (FO1) or 6.48% *w/w* (FO2). The following six milk chocolate treatments were prepared: control (without Prob and FO), probiotics (Prob), fish oil added at 3.24% *w/w* (FO1), fish oil added at 6.48% *w/w* (FO2), Prob + FO1 and Prob + FO2. The chocolate mixture was poured into polycarbonate molds, with a constant shake to avoid bubbles and empty spaces. The leftover mix was removed, and the samples were stored at 4 °C. The chocolates’ probiotic viability was determined by homogenizing 10 g of sample with 90 mL of peptone water (1:9 dilution) in a stomacher for 90 s (IUL Instruments, Spain). Serial dilutions were prepared, and 100 µL of diluted samples were added into the MRS plates by duplicate and incubated for 24 h at 35 °C. The viability of microencapsulated probiotics added to chocolate were differentiated from lactobacilli naturally present in chocolate by performing bacterial counts in the chocolate control (without Prob and FO). Indeed, lactobacilli counts in chocolate control were below the limit of detection.

### 2.4. Fatty Acid Profile

Chocolate fat was extracted following the AOAC 948.22 Soxhlet method, using ethyl ether as the extraction solvent [17,18]. For each formulation, fat extraction (12 g) was performed in triplicate. Fat samples (5 mg) were dissolved in a toluene-hexane mixture (0.6 mL, 1:1 *v/v*). Undecanoic acid (100 ppm, Nu Chek Prep Inc., MA, USA) was added to samples as an internal standard for quantification. Subsequently, samples were transmethylated to fatty acid methyl esters (FAMEs) using methanol-sulfuric acid (1 mL, 93:7 *v/v*) in tightly capped vials placed in a water-bath (80 °C, 60 min). After chilling, the FAMEs were extracted with hexane and volume adjusted to a final volume of 2 mL for gas chromatography analysis.

FAMEs profile was determined on a gas chromatography (GC) system coupled with a flame ionization detector (GC-FID 6850A, Agilent Technologies Inc., Santa Clara, CA, USA). Separation of FAMEs was performed in a fused-silica SP-2380 capillary column (100 m × 0.25 mm i.d., 0.2 µm film thickness; Supelco, Bellefonte, PA, USA). Chromatographic parameters, and FAMEs identification and quantification were performed according to Castillo et al. [19]. Quantification for each compound, and the total amount of fatty acids were calculated by the AOAC method 996.06 [20]. The concentrations of individual fatty acids were expressed as mg of fatty acid per 100 g of fish oil in a fresh weight (FW) basis.

### 2.5. Rheological Analysis

To study chocolate rheology, the flow behavior, frequency sweep, and thermal history variables were analyzed using a stress rheometer (Physica MCR 101, Anton Paar, Germany) fitted with a parallel plate geometry (PP25/S, 24.973 mm diameter). Chocolate samples were prepared before analysis by melting to a constant temperature of 35 °C. The variables flow behavior, frequency sweep, and thermal history were determined as described by Kiumarsi et al. [21], with a modification in the shear rate, which herein increased from 0.1 to 100 s^−1^. Complex values (G*) were obtained and plotted using Equation (1) as follows:

(1) $$G^{*}=\sqrt{{\left(G^{\prime }\right)}^{2}+{\left(G^{\prime \prime }\right)}^{2}}.$$

### 2.6. Texture Analysis, Surface Color, and Water Activity Determinations

Hardness and work penetration (N) of the samples were analyzed using a Texture analyzer TVT 6700 (Perten Instruments, Waltham, MA, USA) equipped with a cylinder probe (height 45 mm, diameter 3 mm). The conditions used were sample height: 8 mm; starting distance from sample to probe: 5 mm; compression; 2 mm; initial speed: 0.5 mm/s; test speed: 0.5 mm/s; retract speed: 10 mm/s; trigger force: 5 g; data rate: 500 pps. The values were expressed as the mean value of five replicates.

Surface color analysis [CIE-LAB, lightness, L*; redness, a*; yellowness, b*; Chroma (C*), and Hue angle (*h*°), and color change ($\Delta E^{*}$)] of chocolates was performed using a Spectrophotometer CM-600d (Konica Minolta, Japan.) with illuminant D65 and 8 mm of sampling aperture. Chroma (C*), hue (h*), $\Delta E^{*}$, and white index (WI) values were calculated using Equations (2)–(4) and Equation (5) respectively, as follows [13]:

(2) $$C^{*}=\sqrt{a^{2}+b^{2}},$$

(3) $$h^{*}=\arctan(a^{*}/b^{*}),$$

(4) $$\Delta E^{*}=\sqrt{{\left(L_{2}-L_{1}\right)}^{2}+{\left(a_{2}-a_{1}\right)}^{2}+{\left(b_{2}-b_{1}\right)}^{2}},$$

(5) $$\mathrm{WI}={\left[{\left(100-L\right)}^{2}+a^{2}+b^{2}\right]}^{1/2}.$$

Water activity ($a_{w}$) determination was performed with an Aqualab CX-2 (Decagon Devices, Washington) at 24 °C using 3.0 g of the samples previously homogenized using a grinder (80350R, Hamilton Beach, USA) for 15 s. The values were expressed as the mean value of three replicates.

### 2.7. Sensory Acceptability Test

A sensory evaluation test was performed to compare the acceptability between the six chocolate formulations. The consumer panel was composed of 119 students and staff from Tecnologico de Monterrey (Monterrey, NL, México). The average age of the consumers was 20 years, and the sample portion was approximately 2 g. The evaluation was based on a hedonic test where appearance, flavor, texture, and overall acceptability parameters were evaluated in a 9-point hedonic scale as follows: 1—“Dislike extremely”, 2—“Dislike very much”, 3—“Dislike moderately”, 4—“Dislike slightly”, 5—“Neither like nor dislike”, 6—“Like slightly” 7—“Like moderately”, 8—“Like very much”, and 9—“Like extremely”. Prior to sensory analysis, the safety of the chocolates was determined according to NOM-186-SSA1/SCFI-2013 [22], where total coliforms and *Salmonella* spp. were analyzed. The plate count method was used in XLD agar for *Salmonella* spp., grown in Tetrathionate and Rappaport Vassiliadis broth, and RVBA agar for total coliforms analysis by duplicate. All chocolates presented <10 CFU/mL for total coliforms fungi and molds and were free of *Salmonella* spp.; thus, all chocolates were safe for human consumption and suitable for sensory evaluations (Ethics ID: CSERMBIGDL-001).

### 2.8. Statistical Analysis

Statistical analyses were performed with data obtained from three replicates of each treatment. Data represent the mean values of three replicates and their standard error. Significant differences between mean values were determined by one-way analysis of variance, followed by the LSD test (*p* < 005), using the JMP statistical software 15.1.0 version (SAS Institute Inc., Cart, NC, USA).

## 3. Results and Discussion

### 3.1. Viability of Probiotics during Microencapsulation and Chocolate-Making Process

To ensure the viability of probiotics and facilitate their use as food ingredient, microencapsulation with protectants is required [23]. Spray-drying technique has been successful to microencapsulate *Lactobacillus* strains such as *L. plantarum* 299v and *L. rhamnosus* GG [23]. Therefore, in the present study, microencapsulation of probiotics was conducted by the spray-drying process using maltodextrin (10% *w/v*) and sodium alginate (2% *w/v*) as preserving agents. The powders’ plate count containing *L. plantarum* 299v and *L. rhamnosus* GG obtained after microencapsulation process was 7 × 10^13^ and 6 × 10^13^ CFU g^−1^, respectively. These results indicate that combining both microencapsulating agents helped maintain the viability of probiotics using spray-drying. Various studies have been carried out using sodium alginate to microencapsulate different strains of *Lactobacillus* and *Bifidobacterium* such as *Lactobacillus acidophilus, Lactobacillus casei, Bifidobacterium bifidum,* and *Bifidobacterium longum* [24] obtaining similar results to the ones reported herein, confirming that sodium alginate helps to maintain the viability of probiotics.

Prior authors have recommended using a combination of alginate with a prebiotic agent to enhance the protection of probiotics in food systems. This combination of microencapsulation agent (i.e., sodium alginate) and prebiotic generates a symbiotic relationship by forming microcrystalline networks that protect bacteria in food systems [24,25]. Therefore, in the present study, maltodextrin was used in combination with sodium alginate to induce a prebiotic effect as previously reported for *Lactobacillus plantarum* [26]. Likewise, maltodextrin has been successfully used as a co-preservative to increase the *Lactobacillus casei* microencapsulation process’s efficiency using spray-drying. Furthermore, maltodextrin helps the probiotic have better protection during storage and decrease the powders’ hygroscopic properties [27].

The obtained powder with microencapsulated probiotics was used as ingredient for the production of chocolates. After the chocolate elaboration process, the probiotic count of the treatments maintained values higher than 1 × 10^6^ CFU per portion (12 g), which has been stated as the minimum viable microorganism count to exert a probiotic effect [28]. Thus, in agreement with a previous report, in which probiotic mixtures of *Lactobacillus spp*. were added to chocolate formulations, results presented herein showed that milk chocolate is an adequate vehicle for the delivery of *Lactobacillus plantarum* 299V and *Lactobacillus rhamnosus* GG [14].

### 3.2. Fatty Acids Profile

Fatty acid profiles and concentrations of fish oil and chocolate formulations were conducted to determine the concentrations of fatty acids from fish oil used, as a source of ω3, and their stability during the chocolate-making process (Table 1). Quantification indicated that the fish oil used as an ingredient contained 34,712.6 ± 57.3 mg of ω3 PUFAs per 100 g. The most abundant ω3 fatty acids identified were DHA (C22:6, 14,122.2 ± 27.0 mg/100 g) and EPA (C20:5, 12,862.1 ± 17.8 mg/100 g).

The total ω3 PUFAs concentration in the control chocolate was 46.0 ± 2.5 mg per 100 g, while EPA and DHA were not detected. Interestingly, the addition of probiotics alone to chocolate increased the content of ω3 by 56.1%, mainly due to the quantification of EPA and DHA. This increment can be attributed to the lipid and fatty acid composition of the cell membrane of probiotics added to chocolate [29]. In addition, lipases produced by probiotics are likely inducing the production and or facilitating the release of free fatty acids from milk fats, further increasing their quantification [30]. This is in agreement with other milk matrices, such as cheese, where microbial lipases can release fatty acids that provide the final flavor [31].

It is well known that oil from cold-water fish is an excellent source of ω3 PUFAs, mainly EPA and DHA [32]. Thus, the addition of fish oil at FO1 and FO2 concentrations to chocolate formulation resulted in a final product with 633.8 ± 43.4 and 1632.2 ± 54.6 mg of ω3 PUFAs per 100 g of sample, respectively, which correspond to 76.0 ± 5.2 mg (FO1) and 195.8 ± 6.5 mg (FO2) of ω3 PUFAs per chocolate portion (12 g).

Fish oil addition in the presence of the probiotic mixture generated a significant decrease (*p* < 0.05) in the ω3 PUFAs content in both treatments (Prob + FO1 and Prob + FO2) as compared with FO1 and FO2. For instance, FO2+Prob treatment showed 16.6% lower ω3 PUFAs content as compared with FO2. The DHA/EPA and total ω3 PUFAs decrease of FO1/FO2 samples compared to the ones combined with probiotics can be attributed to lipid oxidation. A similar observation was reported for milk chocolate added with different EPA/DHA sources where lipid oxidation caused a remarkable loss of essential fatty acids [13]. On the other hand, the change in fatty acids regarding the combination of Prob and FO suggests that the powder with the microencapsulated probiotics contains enzymes likely released from bacteria during the spray-drying process, which modify the fatty acid composition of the product, as it has been reported that lactobacilli produce enzymes capable of modifying PUFAs [33]. Therefore, the decrease of ω3 PUFAs in Prob + O1 and Prob + FO2 treatments could be caused by the conversion of n-3/n-6 PUFAs precursors to saturated fatty acids (stearic acid) by hydrogenation, previously found in *L. plantarum*, causing the slight but significant reduction of PUFAs components [34]. This observation is in agreement with our result, which showed an increase in the content of saturated fatty acids in chocolates added with probiotics at both FO concentrations. However, this hypothesis should be confirmed in further studies.

Although the content of ω3 decreased when probiotics were added to FO1 and FO2 chocolate formulations, the total fatty acids’ content increased (Table 1), where the increases in omega-6 (ω6) concentration took place. This observation could be attributed to *Lactobacillus* sp.’s potential to produce ω6 as previously reported for *L. plantarum*, which is known to produce conjugated linoleic acid (CLA ω6) [35,36].

Many factors should be considered when determining the required daily intake of ω3 PUFAs, such as age, sex, source, diet, and consumer demography [37]. Nowadays, the demand of ω3 PUFAs supplements increased because of their beneficial effects. In this investigation, product development for children was the main focus. Using FAO [38] suggestion for children, ω3 PUFAs intake of 150 mg per day would be required for optimal brain development. Results showed that one serving size of both FO1 and FO1+Prob provided more than 70 mg per portion (47% of daily intake suggestion), whereas FO2 and FO2+Prob provided more than 160 mg per portion (106% of daily intake suggestion). The obtained results were slightly lower than the targeted values (105 mg for FO1 and 210 mg for FO2) according with the formulation, considering the ω3 PUFAs content in FO. This lower content was attributed to production losses in the chocolate manufacturing process [39] where fatty acids of fish oil are highly susceptible to oxidation [40].

### 3.3. Rheological Analysis

**Figure 1:**
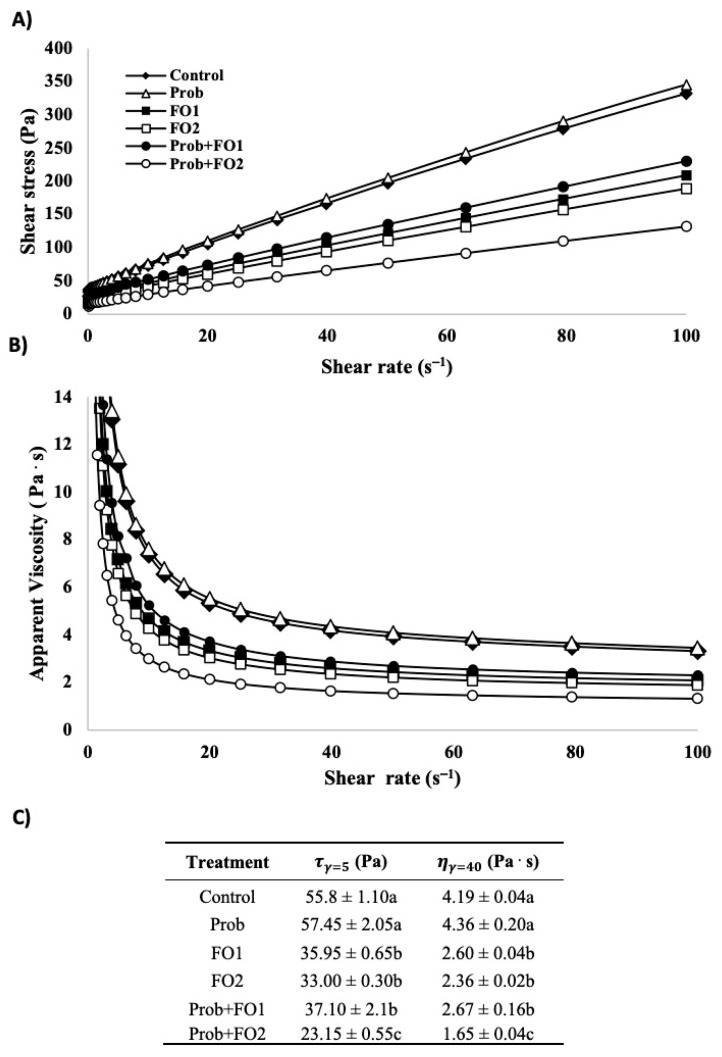
Shear stress (**A**), apparent viscosity, (**B**) and rheological parameters $\tau_{\gamma =5}$ and $\eta_{\gamma =40}$ (**C**) evaluated at 35 °C of chocolate samples added with fish oil (FO) and probiotics (Prob). $\tau_{\gamma =5}$ is the shear stress at shear rate 5 s^−1^ and $\eta_{\gamma =40}$ is the apparent viscosity at shear rate 40 s^−1^. Treatments: Control = FO 0% (*w*/*w*), Prob= Probiotics, FO1 = FO added at 3.24% (*w*/*w*), FO2 = FO added at 6.48% (*w*/*w*). Different letters show significant difference between treatments.

As earlier described, the quality of chocolate can be described by various characteristics, but an essential one is rheology. In chocolate products, the rheology can explain how the different molecules and their molecular structure interact [21]. Chocolates rheology was assessed by its flow behavior, linear viscoelastic measurements, and thermal history parameters. The flow behavior of the chocolate treatments is shown in Figure 1, as the plot of the shear stress (Pa, Figure 1A) and apparent viscosity (Pa·s, Figure 1B) against shear rate (s^−1^) at 35 °C. In addition, rheological parameters of yield stress ($\tau_{\gamma =5},$ shear stress at shear rate 5 s^−1^) and apparent viscosity ($\eta_{\gamma =40}$, apparent viscosity at shear rate 40 s^−1^), are shown in Figure 1C, according to Kiumarsi et al. [21]. Shear rate values were plotted from 0 to 100 s^−1^ for both parameters. In the shear stress (Figure 1A), a shear-thinning behavior was observed for all treatments. This is in agreement with a previous report that evaluated the shear stress of chocolates added with different sources of EPA/DHA [13]. This behavior may be due to the rupture of the structure, causing the collapse of the tridimensional structure of chocolate [21,41]. Furthermore, it can be observed that Prob treatment showed a similar behavior as compared with the control. In addition, results showed that the addition of fish oil decreased the shear stress compared to the control, and a similar behavior was observed for both FO1 (40–190 Pa) and FO2 (40–170 Pa). The treatment that showed an abrupt reduction in shear stress as compared with other samples was the Prob + FO2. The decrease of the shear stress by fish oil addition agrees with previous studies, where milk chocolate added with oil DHA/EPA sources have shown a decrease of the shear rate compared to the control [13].

On the other hand, the apparent viscosity (Pa·s) of the treatments showed a thixotropic behavior (Figure 1B). Higher viscosity values were observed for the control and Prob at a steady shear rate (s^−1^) as compared with FO1 and FO2 treatments added and non-added with probiotics (Figure 1B). As observed for shear stress (Figure 1A), the Prob + FO2 treatment generated a decrease in apparent viscosity to the lowest values. Thixotropic behavior is time-dependent of shear-thinning; this means that the continuous decrease of apparent viscosity generates increased shear stress [41]. This has been previously reported in milk chocolate added with different sources of EPA and DHA [13]. The lower apparent viscosity with FO2 concentration combined with Prob generated a lower chocolate resistance to deformation. In addition, the observation of a reduction of chocolate viscosity by fish oil addition can be supported by previous reports where the percentage of fat in chocolate has been found to decrease the apparent viscosity (Pa·s) and yield stress (Pa) [41]. The results of shear stress and apparent viscosity showed that fish oil addition caused a reduction in chocolates’ viscosity.

Values shown in Figure 1C are useful to measure the viscosity of chocolate and cocoa products, where the shear rate of 5 s^−1^ represents the yield stress of chocolate and viscosity value at a shear rate of 40 s^−1^ to represent the high shear viscosity [16]. The general behavior of the treatments in the *τ* and *η* parameters (Figure 1C) and the plots in Figure 1A,B are similar. No significant difference is shown between the control an Prob treatment. In addition, FO addition at both concentrations generated a significant decrease in both shear stress and apparent viscosity parameters in a 5 and 40 s^−1^ shear rates, respectively. Furthermore, the combination of FO2 and Prob also generated an abrupt and significant decrease as compared to all treatments.

**Figure 2:**
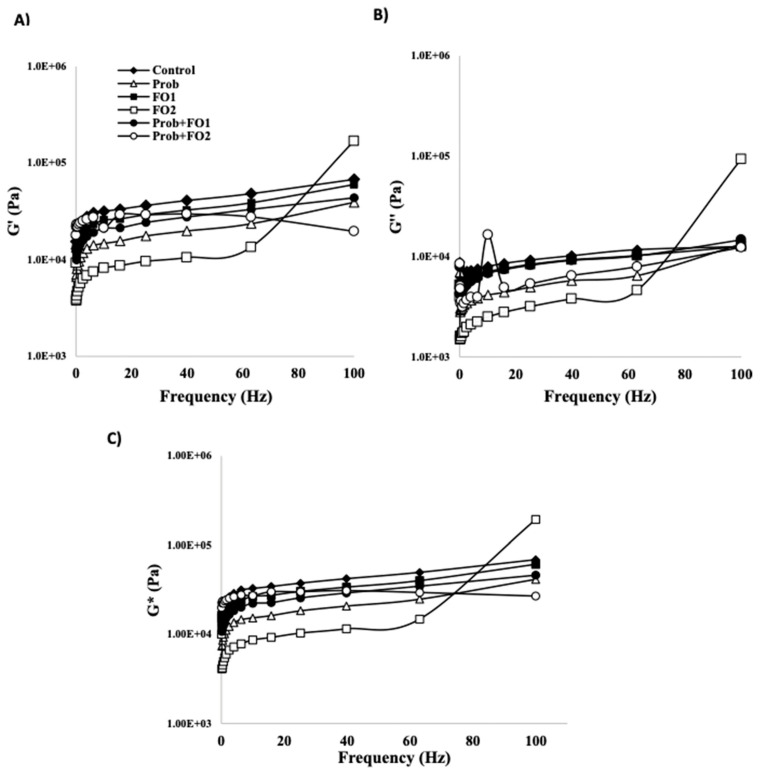
Changes in storage modulus G′ (**A**), loss modulus G″ (**B**), and complex modulus G* (**C**) in the frequency sweep test of chocolate samples (Temperature 35 °C). Treatments: Control, Prob = Probiotics, FO1 = fish oil added at concentration 3.24% (*w*/*w*), FO2 = fish oil added at concentration 6.48% (*w*/*w*).

To determine the linear viscoelastic properties of chocolate formulations, a stress sweep test was performed where the storage modulus (G′), loss modulus (G″) and complex modulus (G*) were plotted at a 1 Hz frequency (Figure 2). Storage modulus (G′) is shown in (Figure 2A) were the control showed the highest values. Prob treatment storage modulus decreased as compared with the control, and showed lower values than FO1, Prob + FO1, and Prob + FO2. Both fish oil treatments (FO1 and FO2) showed lower G′ values than the control, and only FO1 showed higher values than Prob treatment. Likewise, the lowest values for FO2 concentration can be observed. Both Prob + FO1 and Prob + FO2 decreased G′ compared to the control and showed higher values than probiotics treatment. The values of G′ closer to the control were FO1 and Prob + FO1 treatments. Likewise, it can be observed that the lowest values of G′ can be found for FO2 treatment. Loss modulus (G″) values are shown in Figure 2B, and as observed, the behavior was similar to the storage modulus (G′). Analysis with a stress sweep test can provide valuable information on the structural strength of chocolate.

Complex modulus (G*) is shown in (Figure 2C), this module is related to storage modulus (G′) and loss modulus (G″), obtaining its value with a G′/G″ relationship. The value of G* provide information about the rigidity of the chocolate structure [21]. This means that having higher values of G′ compared to G″ results in higher values of G*, which depends on the formulation. Therefore, when G′ is higher than G″, higher values of G* are obtained, indicating that chocolate presents greater rigidity. High G* values have also been associated with a high level of interaction between the forces of the chocolate particles [42]. Likewise, in (Figure 2C) it can be observed that G*s values of the treatments were higher than the control in 1–60 Hz frequency, and thus it can be concluded that chocolates tend to show a solid-like behavior [21]. A solidification behavior was previously reported by Taghizadeh et al. [43] in the formulation of a chocolate mousse added with probiotics where G′ was above G″. G′ values closer to the control were obtained for FO1 and Prob + FO1 treatments, which were associated with higher rigidity.

**Figure 3:**
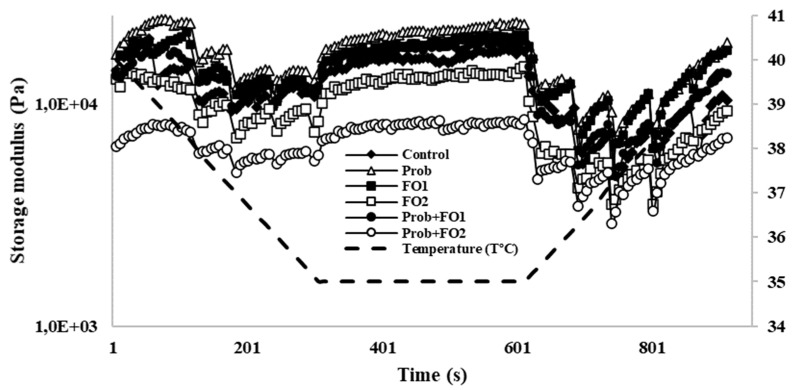
Effect of heating and cooling on the rheological behavior of chocolate samples. Change of temperature from 6 to 40 °C (f = 1 Hz). Treatments: Control, Prob = Probiotics, FO1 = fish oil added at concentration 3.24% (*w*/*w*), FO2 = fish oil added at concentration 6.48% (*w*/*w*).

To determine the effect of heating and cooling on the behavior of the different chocolate treatments, a thermal history analysis was determined (Figure 3). Prob treatment presented higher storage modulus values (Pa) as compared with the control. The addition of FO1 showed a behavior similar to the control. When fish oil concentration changed to FO2, the storage modulus decreased. In addition, both Prob + FO1 and Prob + FO2 generated an increase in storage modulus compared to the control. Furthermore, an increase in storage modulus was observed when the temperature decreased to 35 °C. On the other hand, there was similar behavior of the FO1 to the control, followed by Prob + FO1. Treatments with the lowest storage modulus values were FO2 and Prob + FO2. Both treatments with FO2 concentration did not behave stably. The behavior of Prob + FO2 treatment was further away from the control, thus their rheological characteristics were not the most adequate at the time of the simulation of chocolate manufacturing. This decrease may be due to the higher amount of oil in the chocolates, because it has been reported that the fat content of chocolate can potentiate changes in the thermal ramps [15]. Melting properties are critical to evaluate the quality of chocolate samples because of their impact on appearance and stability.

As a consequence, consumer acceptability may be influenced by the loss of glossy appearance and the fat bloom formation [15]. On the other hand, both Prob and Prob + FO1 treatments were the ones with a behavior closest to the control. In addition, an increase in the storage modulus was observed when the temperature decreased to 35 °C, and this could be explained due to fat crystals generated by heat and the recovery of chocolate structure [21].

According to the rheological analysis results, it can be concluded that when comparing all chocolate treatments with fish oil, the Prob + FO1 was the most similar to the control for flow behavior and thermal analysis. In addition, FO1 and Prob + FO1 presented a similar behavior to control. Therefore, Prob + FO1 chocolate is the treatment with greater viability because of its similar rheological behavior as compared with the control.

### 3.4. Surface Color, Water Activity, and Texture Analysis

Surface color parameters L*, a*, b*, C*, *h*° (hue), ΔE* (color change), WI (white index), and water activity (*a_w_*) values for the different chocolate treatments are shown in Table 2. Regarding the luminosity parameter (L*), values ranged from 9.98 to 19.2 and significant differences (*p* < 0.05) were detected between the control and all the treatments. The addition of Prob and FO at the two concentrations generated a decrease in L* value, where FO2 showed the lowest value compared with the control. Since the luminosity value indicates how it goes from 0 (black) to 100 (white), the addition of fish oil and probiotics caused a decrease in light intensity compared to the control. Furthermore, ΔE* values ranged from 2.81 to 9.13, indicating that FO or Prob added either individually or combined, generates a significant change in the instrumental color values as compared with the control. These results agreed with the report of Foong et al. [14], where the incorporation of *L. plantarum* to dark chocolate formulation decreased L* values compared to the control chocolate. Likewise, the change in colors when adding the probiotics can be attributed to the form added to the formulation, since they were incorporated as microencapsulated white powder. Therefore, this could generate a lighter shade in the Prob + FO1 and Prob + FO2 treatments than pure fish oil. Additionally, the addition of fish oil generated a decrease of L* values compared to the control, as shown in Konar et al. [13], where the addition of different sources of EPA/DHA rich oils decreased the values compared to the control. Therefore, the milk chocolate added with FO2 resulted in the darkest chocolate compared to all treatments. An observation demonstrated that the probiotics and fish oil addition, at both concentrations, reduced luminosity (L*) compared with the control.

On the other hand, a* values showed changes from 3.93 to 4.60. The control showed the highest values, and a significant difference was only observed with the Prob + FO1 treatment. Furthermore, no significant differences were observed for the b* and Chroma (C*) values between treatments. The values obtained for *h*° ranged from 0.77 to 0.85, where the control showed the lowest value, and no significant difference was detected between treatment. The positive values in a* and b* represented that the color changes could be observed from predominant red and yellow colors. The values of both parameters are similar to those obtained in a study of the comparison of two different chocolates, where the pure milk chocolate control showed a* and b* values of 5.59 and 5.38, respectively [44]. In this case, both a* and b* ranges match with regular milk chocolate’s values. The C* values measure the color’s purity, measuring the distance from a gray level with the same brightness where C* = 0 [45]. Recent studies on chocolate products added with EPA and DHA sources resulted in the range 9.55–11.7 for C* in milk chocolate, 27.0–27.7 for white chocolate, and 7.45–8.09 for dark chocolate [13,46,47]. Their observations suggest that the values obtained in this experiment for C* tend towards the lower values of dark chocolate. On the other hand, the hue angle (*h*°) ranges from 0° (red) through 90° (yellow), 180° (green), 270° (blue), and back to 0° [45]. The values found in this study are close to the range of values found by Konar et al. [13] in their milk chocolate bar added with EPA/DHA, whose *h*° values ranged from 0.77–0.81.

A crucial parameter to consider in the chocolate industry is fat blooming, because it affects the quality of the appearance and texture, and thus the consumer satisfaction level. Furthermore, the development of fat blooming can also generate changes in the microstructure, appearance, and melting characteristics. This problem occurs when there is incorrect cooling during the tempering process or inadequate storage conditions; therefore, this problem is usually evaluated using the white index (WI) parameter. Chocolate’s gray-whitish appearance indicates an increase in the WI [13,46,47,48], which makes the product undesirable for consumers, since this defect in appearance is associated with expired chocolate products, decreasing the product sales [13,46,48]. In this context, it was considered relevant to determine WI values of chocolates added with FO and Prob, to ensure the correct manufacture of the product, and to determine if the addition of the active ingredients generated a significant change of the WI values. The WI values ranged from 9.76 to 18.85, and the control treatment presented the highest value and FO2 the lowest. The addition of probiotics, fish oil (FO1 and FO2) and the combination generated a decrease of WI values compared with the control. The treatment closer to the control was Prob + FO1 with a 13.64 value. The higher the fish oil concentration, the lower the WI values. This can be supported by the values for dark chocolate obtained by Toker et al. [47], where the WI values decreased from the control concerning the treatment with fish source (EPA/DHA). However, the addition of only probiotics to the chocolate decreased the WI values compared to the control but combined with fish oil values incremented compared to those with fish oil only. This change can be attributed to the recrystallization of lipids influenced by probiotics previously observed by Silva et al. [49] in developing semisweet chocolate containing probiotics.

Water activity ($a_{w}$) values of chocolate treatments ranged from 0.44 to 0.50; which is considered a critical parameter to assess the stability and quality because it specifies the minimum limits of available water for bacterial growth. It has been reported to be inversely correlated to fat content [13,46]. The growth of fungi begins at an $a_{w}$ of 0.75, yeasts at 0.8, and bacteria at 0.9 values of $a_{w}$ [50]. Results did not exceed the limit $a_{w}$ values for growth of fungi and bacteria; thus, all the treatments were within the permissible limit. Except for Prob + FO2, decreases in $a_{w}$ values were observed in all treatments. This decrease can be due to the effect of the concentration of PUFAs contained in fish oil source. The lower $a_{w}$ values observed with fish oil were added to the formulations, and can be related with encapsulation of the ingredients by the fish and a decrease of electric charges that can interact with water molecules when there is a higher degree of unsaturation in a fatty acid [51]. Furthermore, the decrease of $a_{w}$ in Prob treatment may be due to maltodextrin presented as a microencapsulate agent. Maltodextrin is used as an $a_{w}$-lowering agent that reduces the final water activity of products [52]. In addition, sodium alginate may reduce water activity of foods because of sodium ions’ ability to associate with molecules of water [53].

Texture is an important parameter to assess the quality of chocolates in the confectionery industry. The hardness is one of the parameters used to analyze a product’s texture, being one of the most important since it is used as an indicator of the quality of a product towards its consumer [13]. Texture values of chocolate formulations are shown in Table 3. The values of hardness ranged from 925.8 N to 3095.8 N. The addition of probiotics alone did not affect the hardness value of the chocolate. On the other hand, fish oil addition at FO1 and FO2 concentrations generated a decrease in hardness. The addition of fish oil and probiotics generated a decrease in the hardness compared to the control; therefore, the lowest value was observed in the Prob + FO2 treatment. A decrease in the chocolate hardness, with fish oil in the formulation, has been previously observed in the development of milk, black and white chocolates added with different sources of EPA/DHA [13,47]. It has been reported that there is a relationship between the hardness of chocolates with the fatty acid profile. For instance, the high level of PUFAs decreases hardness due to their lower melting temperature than saturated fatty acids [47]. These results agree with the fatty acids profile shown in Table 1, where chocolates added with fish oil (FO1 and FO2) showed higher PUFAs levels.

Work of penetration (N) is the work that is obtained by the area under the force curve and is also associated with the maximum force for penetration [54]. The work of penetration values ranged between 1475 N and 3513 N (Table 3). Significant differences (*p* < 0.05) can be observed between the control and the treatments with FO1 and FO2. Likewise, results showed the lowest work of penetration values for FO2 and Prob + FO2 treatments. Results indicated that FO2 and Prob + FO2 treatments broke with less force.

### 3.5. Sensory Acceptability Test of Chocolates Added with Fish Oil and Probiotics

The results for the sensory acceptability test of milk chocolate samples added with fish oil and probiotics is shown in (Table 4). A hedonic sensory test using a 9-point scale was performed, with different acceptability, evaluating different parameters such as appearance, taste, texture, and overall acceptability. There were no significant differences (*p* > 0.05) between the acceptability of Prob and the control treatment in the parameters evaluated. Similar results were obtained by Silva et al. [49] where the acceptability of semisweet chocolate formulation was not affected when added with probiotics.

The addition of fish oil at FO1 concentration did not modify the appearance, texture, and overall acceptability of chocolate as compared to the control; however, the acceptability of flavor slightly decreased, showing values in the acceptable range (>6). Both FO1 and Prob + FO1 treatments maintained adequate overall acceptability values (>7). However, chocolates added with fish oil at FO2 levels showed a significant decrease in the product’s sensory acceptability (Table 4). These results agree with a previous report, where the addition of EPA and DHA sources in milk chocolate showed a significant effect on the product’s sensory properties [13]. In this study, both FO1 and Prob + FO1 chocolate formulations were promising, contrasting the previous studies by Konar et al. [13], where milk chocolate formulations added with EPA/DHA did not exceed the recommended scores with 6.00 ± 1.09 for overall acceptability. It is likely that in this study overall acceptability of FO1 was adequate, because EPA/DHA concentration was lower with 451.7 mg (EPA/DHA per 100 g) than Konar et al. [13], with 672.4 mg (EPA/DHA per 100 g). Considering Konar et al. [13], overall acceptability for chocolate added with EPA/DHA from the fish source (6.00 ± 1.09) showed closer results to FO2 (5.57 ± 0.13), which showed a concentration of 1,211 mg EPA/DHA per 100 g of chocolate. In this case, despite the lower scores of FO1 and Prob + FO1, if the product’s benefits are revealed to consumers, the acceptability and willingness to pay for the product could be increased [55]. Finally, chocolate treatments with FO2 and Prob + FO2 were not preferred at all by the panelists, and thus these formulations are not an option for commercialization. A gender segmentation analysis of acceptability data was further performed to better understand the behavior of consumers. The percentage of females and males that graded the overall acceptability of chocolates with a value equal or greater than 6 was determined. From this segmentation analysis, it was determined that the percentage of male participants grading the product with overall acceptability equal or higher than 6 was higher for all treatments, indicating that gender is an important driver of chocolate consumer’s preferences, as the female participants were more critical.

## 4. Conclusions

Based on the results obtained in this research, it is feasible to obtain a chocolate formulation added with probiotics and ω3 PUFAs, with adequate sensory acceptability from consumers. The addition of fish oil combined with probiotic strains showed high concentrations of ω3 fatty acids for Prob + FO1 (599.6 ± 41.5 mg ω3 per 100 g) and Prob + FO2 (1360.3 ± 72.5 mg ω3 per 100 g), maintaining 1 × 10^6^ CFU per portion. Therefore, the combination may potentiate brain and cognitive functions connecting the brain-gut axis.

An adequate candidate for commercialization was the Prob + FO1 treatment considering rheological characteristics, color, texture, and its potential benefits. It was possible to develop a bar of milk chocolate added with fish oil and probiotics, maintaining its stability. It is essential to consider the concentration of fish oil added to maintain the chocolate’s physicochemical qualities. Although the chocolate formulation FO1 showed acceptable values, if higher levels of ω3 were desired in the chocolate formulation, it is suggested to find alternative sources or presentations of fish oil such as flaxseeds and microalgae oil, incorporating co-encapsulation with probiotics. Further research should consider performing clinical studies for the validation of the nutraceutical functionality of the chocolate formulation added with ω3 PUFAs and probiotics on the cognitive development of school-age children. Likewise, it would be relevant to determine the shelf-life stability of chocolates as well as their chemical composition before going to the market. Moreover, further studies should consider evaluating the viability of microencapsulated probiotic bacteria during storage.

## Figures and Tables

**Table 1:** Fatty acid profile of fish oil source and milk chocolate samples added with probiotics and fish oil.

Fatty Acid	Fish Oil	Chocolate Samples (mg Fatty Acid per 100 g)					
		Control	Prob	FO1	FO2	Prob + FO1	Prob + FO2
Octanoic Acid (C8:0)	183.6 ± 14.6	93.7 ± 9.9 ab	91.2 ± 3.1 ab	87.3 ± 6.5 b	89.5 ± 5.2 ab	109.6 ± 4.9 a	96.2 ± 8.8 ab
Decanoic acid (C10:0)	N.D.	128.9 ± 1.4 abc	128.1 ± 5.6 abc	122.7 ± 1.9 bc	115.7 ± 1.9 bc	135.0 ± 4.8 ab	140.2 ± 9.3 a
Lauric acid (C12:0)	98.3 ± 1.7	116.5 ± 4.2 ab	120.5 ± 5.4 a	117.0 ± 2.3 ab	107.1 ± 2.4 b	126.4 ± 4.4 a	129.3 ± 5.3 a
Myristic acid (C14:0)	8375.8 ± 22.2	395.2 ± 11.5 c	437.6 ± 29.2 c	549.0 ± 17.4 b	770.7 ± 16.0 a	565.5 ± 24.1 b	771.3 ± 40.4 a
Pentadecanoic acid (C15:0)	759.4 ± 6.9	48.8 ± 1.7 d	51.8 ± 2.0 cd	60.0 ± 2.1 bc	78.0 ± 1.6 a	63.7 ± 2.5 b	82.8 ± 5.5 a
Palmitic acid (C16:0)	16,842.4 ± 62.0	5541.8 ± 167.0 c	5911.3 ± 298.2 bc	5887.6 ± 118.2 bc	5767.2 ± 128.5 bc	6312.8 ± 280.5 ab	6691.5 ± 285.9 a
Heptadecanoic acid (C17:0)	683.0 ± 6.3	62.0 ± 5.4 d	68.8 ± 3.4 cd	77.1 ± 2.5 bc	88.4 ± 1.9 ab	79.4 ± 3.8 bc	95.4 ± 4.6 a
Stearic acid (C18:0)	3283.4 ± 9.5	6281.7 ± 193.1 b	6658.4 ± 327.7 a	6414.8 ± 115.8 ab	5743.6 ± 137.0 b	6919.3 ± 316.2 a	6946.7 ± 277.7 a
Arachidic acid (C20:0)	178.6 ± 2.7	188.7 ± 5.5 bc	200.0 ± 10.2 ab	194.0 ± 3.5 abc	176.1 ± 4.4 c	208.8 ± 9.5 ab	212.1 ± 8.6 a
Behenic acid (C22:0)	149.8 ± 3.0	30.7 ± 1.0 c	33.6 ± 2.1 bc	32.8 ± 0.8 bc	35.5 ± 0.8 abc	37.5 ± 2.4 ab	40.2 ± 3.8 a
Lignoceric acid (C24:0)	165.9 ± 1.8	21.4 ± 1.0 c	23.7 ± 0.6 bc	21.2 ± 1.1 c	25.1 ± 1.1 b	25.5 ± 0.6 b	29.9 ± 1.8 a
Myristoleic acid (C14:1)	59.0 ± 2.2	35.4 ± 1.5 bc	36.7 ± 0.6 abc	36.0 ± 1.2 bc	34.0 ± 1.2 c	39.4 ± 2.3 ab	41.9 ± 2.6 a
Palmitoleic acid (C16:1)	11,325.6 ± 19.1	109.3 ± 3.1 d	139.3 ± 19.1 d	311.0 ± 18.8 c	649.5 ± 12.2 a	300.1 ± 14.3 c	565.7 ± 34.4 b
Oleic Acid (C18:1)	4907.2 ± 26.6	5845.3 ± 148.1 ab	6160.2 ± 347.3 ab	6065.5 ± 100.7 ab	5496.3 ± 113.1 b	6452.7 ± 277.8 a	6522.1 ± 250.1 a
Vaccenic acid (C18:1)	2906.6 ± 12.9	73.8 ± 1.4 d	83.0 ± 6.5 d	122.4 ± 4.7 c	206.6 ± 4.2 a	123.6 ± 6.0 c	186.6 ± 8.2 b
Eicosenoic acid (C20:1)	498.5 ± 3.2	N.D.	N.D.	16.0 ± 1.6 c	30.6 ± 0.6 a	17.6 ± 1.6 c	26.1 ± 2.1 b
Nervonic acid (C24:1)	399.5 ± 1.6	N.D.	N.D.	N.D.	21.5 ± 2.7 a	N.D.	16.7 ± 0.7 b
Linoleic acid (C18:2)	1842.2 ± 47.9	622.5 ± 17.4 c	635.3 ± 31.6 bc	652.9 ± 11.7 bc	625.8 ± 16.3 c	704.8 ± 31.1 ab	732.6 ± 27.7 a
Gamma Linolenic acid (C18:3)	268.3 ± 5.0	N.D.	N.D.	N.D.	15.2 ± 0.6 a	N.D.	13.5 ± 0.5 b
Alpha Linolenic acid (C18:3)	2009.1 ± 6.7	46.0 ± 2.5 c	49.3 ± 2.4 c	81.0 ± 3.4 b	134.2 ± 4.1 a	83.6 ± 5.7 b	125.1 ± 6.5 a
Stearidionic acid (C18:4)	3458.1 ± 18.5	N.D.	N.D.	53.3 ± 3.9 c	181.0 ± 17.5 a	54.9 ± 3.3 c	131.3 ± 6.7 b
Eicosadienoic acid (C20:2)	458.7 ± 1.8	N.D.	N.D.	8.3 ± 0.2 b	23.4 ± 4.8 a	10.5 ± 0.5 b	19.5 ± 1.4 a
Homo-gamma-linolenic acid (C20:3)	282.5 ± 1.4	N.D.	N.D.	8.4 ± 0.4 b	16.2 ± 0.8 a	10.1 ± 0.7 b	17.0 ± 1.0 a
Dihomogamma linolenic acid (C20:3)	372.9 ± 22.5	N.D.	N.D.	11.0 ± 1.3 b	21.0 ± 1.8 a	10.8 ± 1.1 b	18.4 ± 1.7 a
Arachidonic acid (C20:4)	851.2 ± 9.1	N.D.	N.D.	31.7 ± 4.0 b	44.6 ± 2.4 a	21.3 ± 2.1 c	41.7 ± 1.9 a
Eicosapentaenoic acid (C20:5)	12,862.1 ± 17.8	N.D.	12.6 ± 3.0 d	214.5 ± 19.2 c	571.0 ± 16.9 a	199.9 ± 8.9 c	460.4 ± 22.4 b
Docosapentaenoic acid n-6 (C22:5)	331.1 ± 21.1	N.D.	N.D.	N.D.	18.8 ± 0.8 a	N.D.	20.5 ± 1.9 a
Docosapentaenoic acid n-3 (C22:5)	1888.1 ± 25.5	N.D.	N.D.	36.8 ± 1.4 c	85.0 ± 1.9 b	41.8 ± 5.0 c	104.0 ± 11.9 a
Docosahexaenoic acid (C22:6)	14,122.2 ± 27.0	N.D.	9.9 ± 1.4 d	237.2 ± 22.1 c	640.0 ± 18.8 a	208.6 ± 22.0 c	521.0 ± 26.0 b
Total ω-3	34,712.6 ± 57.3	46.0 ± 2.5 d	71.8 ± 5.2 d	633.8 ± 43.4 c	1632.2 ± 54.6 a	599.6 ± 41.5 c	1360.3 ± 72.5 b
Total ω-6	4034.0 ± 29.6	622.5 ± 17.4 c	635.3 ± 31.6 c	701.2 ± 13.5 bc	744.0 ± 22.5 b	746.7 ± 34.3 b	844.8 ± 33.5 a
Saturated fatty acids (SFA)	30,720.2 ± 103.6	12,909.4 ± 400.6 c	13,724.9 ± 675.2 abc	13,563.7 ± 264.9 bc	12,997.0 ± 298.1 bc	14,583.4 ± 652.9 ab	15,235.6 ± 651.0 a
Monounsaturated fatty acids (MUFA)	20,096.3 ± 55.2	6063.7 ± 153.8 c	6419.1 ± 359.7 bc	6550.9 ± 124.4 bc	6438.3 ± 131.4 bc	6933.4 ± 301.8 ab	7359.1 ± 296.8 a
Polyunsaturated fatty acids (PUFA)	38,746.6 ± 45.8	668.5 ± 19.6 c	707.1 ± 34.0 c	1335.1 ± 51.9 b	2376.1 ± 74.4 a	1346.3 ± 72.1 b	2205.0 ± 105.5 a
Total fatty acids	89,563.1 ± 189.0	19,641.6 ± 574.0 c	20,851.2 ± 1064.8 bc	21,449.7 ± 432.9 bc	21,811.5 ± 502.5 bc	22,863.2 ± 1022.2 ab	24,799.7 ± 1052.5 a

N.D., none detected; different letters show the significance between treatments (*p* < 0.05), all experiments were performed in triplicate. All data are presented as means ± S.D. Treatments: Control, Prob = Probiotics, FO1 = fish oil added at concentration 3.24% (*w*/*w*), FO2 = fish oil added at concentration 6.48% (*w*/*w*).

**Table 2:** Instrumental color CIE LAB values and water activity (*a_w_*) of milk chocolate added with probiotics and fish oil.

Treatments	Color Properties							*a_w_*
	L*	a*	b*	C*	*h*°	ΔE*	WI	
Control	19.1 ± 2.12 a	4.60 ± 0.04 a	4.42 ± 0.04 a	6.38 ± 0.01 a	0.77 ± 0.01 b	0	18.85 ± 2.11 a	0.50 ± 0.03 a
Prob	12.5 ± 1.25 bc	4.12 ± 0.24 ab	4.36 ± 0.29 a	6.00 ± 0.35 a	0.81 ± 0.02 a	6.68 ± 1.25 abc	12.25 ± 1.25 bc	0.45 ± 0.00 b
FO1	13.3 ± 0.33 bc	4.26 ± 0.22 ab	4.54 ± 0.41 a	6.23 ± 0.45 a	0.81 ± 0.02 a	5.90 ± 0.32 bc	13.03 ± 0.35 bc	0.44 ± 0.00 b
FO2	9.98 ± 0.67 c	4.22 ± 0.05 ab	4.72 ± 0.09 a	6.33 ± 0.10 a	0.84 ± 0.00 a	9.13 ± 0.67 a	9.76 ± 0.68 c	0.46 ± 0.00 b
Prob + FO1	13.83 ± 0.61 b	3.93 ± 0.25 b	4.09 ± 0.33 a	5.67 ± 0.41 a	0.80 ± 0.01 ab	5.35 ± 0.64 c	13.64 ± 0.59 b	0.44 ± 0.00 b
Prob + FO2	11.20 ± 0.87 bc	4.31 ± 0.28 ab	4.87 ± 0.41 a	6.50 ± 0.49 a	0.85 ± 0.01 a	7.94 ± 0.89 ab	10.96 ± 0.90 bc	0.47 ± 0.00 ab

Different letters show significant difference between treatments (*p* < 0.05).; L*, brightness from black (0) to white (100); a*, from green (−) to red (+); b*, from blue (−) to yellow (+); C*, chroma; *h*°; hue angle; Δ*E*, color change; WI, white index; $a_{w}$, water activity. All data are presented as means ± S.D. Treatments: Control, Prob = Probiotics, FO1 = fish oil added at 3.24% (*w*/*w*), FO2 = fish oil added at 6.48% (*w*/*w*).

**Table 3:** Texture parameters values of milk chocolate added with probiotics and fish oil.

Treatment	Hardness (N)	Work of Penetration (N)
Control	2959.2 ± 232.7 a	3513 ± 245.1 a
Prob	3095.8 ± 136.1 a	3147.6 ± 143.0 ab
FO1	2271.4 ± 85.6 b	2906.6 ± 141.2 b
FO2	1430.8 ± 75.5 c	1903.8 ± 104.3 c
Prob + FO1	2049.8 ± 155.1 b	3069.6 ± 345.1 ab
Prob + FO2	925.8 ± 49.5 d	1475 ± 102.4 c

Different letters significant difference between treatments (*p* < 0.05). All data are presented as means ± S.D. Treatments: Control = fish oil 0%, Prob = Probiotics, FO1 = fish oil added at 3.24% (*w*/*w*), FO2 = fish oil added at 6.48% (*w*/*w*).

**Table 4:** Sensory acceptability values of chocolate samples added with fish oil and probiotics.

Parameters	Treatments					
	Control	Prob	FO1	FO2	Prob + FO1	Prob + FO2
Appearance	7.54 ± 0.11 a	7.46 ± 0.16 a	7.34 ± 0.12 abc	7.03 ± 0.13 bc	7.37 ± 0.12 ab	6.92 ± 0.17 c
Flavor	8.68 ± 0.11 a	7.58 ± 0.22 ab	7.25 ± 0.09 bc	5.26 ± 0.16 d	7.00 ± 0.16 c	5.04 ± 0.21 d
Texture	7.59 ± 0.13 a	7.58 ± 0.19 a	7.57 ± 0.12 a	6.38 ± 0.14 b	7.31 ± 0.14 a	6.06 ± 0.21 b
Overall acceptability	7.83 ± 0.10 a	7.59 ± 0.21 a	7.42 ± 0.11 ab	5.57 ± 0.13 c	7.09 ± 0.14 b	5.46 ± 0.20 c

Different letters show significant difference between treatments (*p* < 0.05). All data are presented as means ± S.D. Values represent the mean of the evaluation of 119 chocolate consumers using a hedonic test (1–9); where: 1—”Dislike extremely”, —“Dislike very much”, 3—“Dislike moderately”, 4—“Dislike slightly”, 5—“Neither like nor dislike”, 6—“Like slightly” 7—“Like moderately”, 8—“Like very much”, and 9—“Like extremely”. Treatments: Control= fish oil 0%, Prob = Probiotics, FO1 = fish oil added at 3.24% (*w*/*w*), FO2 = fish oil added at 6.48% (*w*/*w*).

## Data Availability

Not applicable.
